# Impact of Heat Stress on Carcass Traits, Meat Quality, and Nutritional Value in Monogastric Animals: Underlying Mechanisms and Nutritional Mitigation Strategies

**DOI:** 10.3390/foods14091612

**Published:** 2025-05-02

**Authors:** José A. M. Prates

**Affiliations:** 1 CIISA—Centro de Investigação Interdisciplinar em Sanidade Animal, Faculdade de Medicina Veterinária, Universidade de Lisboa, Av. da Universidade Técnica, 1300-477 Lisboa, Portugal; japrates@fmv.ulisboa.pt; 2 Associate Laboratory for Animal and Veterinary Sciences (AL4AnimalS), Av. da Universidade Técnica, 1300-477 Lisboa, Portugal

**Keywords:** heat stress, monogastric animals, carcass traits, meat quality, nutritional strategies, oxidative stress

## Abstract

This review examines the impact of heat stress (HS) on carcass traits, meat quality, and nutritional composition in monogastric animals, specifically poultry and swine, and evaluates targeted nutritional strategies for mitigation. With rising global temperatures and intensified heat waves, HS has emerged as a key threat to animal welfare, production efficiency, and meat quality. Physiological disturbances induced by HS, including oxidative stress, protein denaturation, mitochondrial dysfunction, and hormonal imbalances, contribute to reduced carcass yield, muscle degradation, and inferior sensory attributes such as tenderness, juiciness, and flavour. HS also diminishes the nutritional value of meat by depleting essential amino acids, polyunsaturated fatty acids, and antioxidant micronutrients. This review highlights nutritional interventions, including antioxidant supplementation (e.g., vitamin E, selenium, polyphenols), osmolytes (e.g., betaine, taurine), probiotics, prebiotics, and optimised energy-to-protein ratios, as promising tools to enhance thermotolerance and meat quality. Emerging feed additives such as phytochemicals also show potential for protecting muscle integrity and improving oxidative stability. Given species-specific responses and production system variability, integrating these dietary approaches with stage-specific management is essential for resilience under climate stress. Future research should focus on the precision nutrition, biomarker identification, and validation of synergistic nutritional strategies that safeguard performance and meat quality in monogastric production systems.

## 1. Introduction

Global warming and the increasing frequency of extreme weather events have fundamentally reshaped modern agriculture [1]. Among the environmental stressors affecting animal production, heat stress (HS) has emerged as one of the most significant challenges, particularly in monogastric production systems. Climate change has led to more frequent and intense heat waves, pushing ambient temperatures beyond historical norms, which has had significant consequences for both natural ecosystems and agricultural productivity [2]. This temperature rise not only disrupts the balance of ecosystems but also places substantial pressure on animal production systems. HS directly impairs animal growth, feed conversion efficiency, reproductive performance, and overall health, thereby reducing production efficiency and economic returns [3]. Elevated temperatures compromise animal welfare and production efficiency across all livestock systems.

However, monogastric species such as pigs and poultry are particularly vulnerable due to their limited thermoregulatory capacity and high metabolic rates, making them a critical focus in the context of climate-induced HS [4]. Specifically, it leads to reduced carcass weight, lower dressing percentage, increased fat deposition, and an altered fat-to-muscle ratio, all of which negatively impact meat quality and market value [5,6]. These effects are especially pronounced in intensive production systems where animals are exposed to sustained thermal loads without adequate environmental or nutritional support. With global demand for high-quality animal protein increasing steadily, understanding and addressing the impacts of HS is critical for ensuring food security and sustainable agricultural development. Developing climate-resilient animal production systems and incorporating effective mitigation strategies will be essential to maintaining productivity and meeting growing consumer demand [2].

Although the term ‘monogastric animals’ includes a broad range of species, this review focuses specifically on poultry and swine, which account for the vast majority of global monogastric livestock production. These species are not only the most economically important, but also the most thoroughly researched regarding the physiological effects of HS and the efficacy of nutritional mitigation strategies. These animals are particularly vulnerable to HS due to their limited ability to dissipate heat through sweating or panting, combined with high metabolic rates in intensive production systems [7]. In swine, HS alters thermoregulatory and endocrine pathways, leading to increased cortisol secretion, decreased feed intake, and reduced nutrient absorption [8]. Similarly, in poultry, elevated ambient temperatures disrupt thyroid hormone activity, increase oxidative stress, and compromise intestinal integrity, ultimately impairing growth performance, immune function, and carcass quality [9].

These physiological disruptions are central to the decline in meat quality and nutritional value observed under HS conditions. When environmental temperatures exceed the animals’ thermoneutral zone, the balance between heat production and heat dissipation is disrupted, leading to physiological disturbances that impair growth performance, weaken immune function, and deteriorate overall meat quality [10]. For adult poultry, the thermoneutral zone ranges from 18 to 24 °C for broilers and 18 to 22 °C for laying hens [11]. For adult swine, the thermoneutral zone is 18–24 °C for growing and finishing pigs and 16–22 °C for gestating and lactating sows [12]. HS reduces feed intake and induces metabolic imbalances, which not only diminish growth rates but also affect the physical and chemical properties of muscle tissue [13]. In pigs, HS has been shown to disrupt nutrient metabolism, including protein, lipid, and carbohydrate utilisation, which leads to reduced muscle development and compromised meat quality [14]. To ensure appropriate mitigation strategies, it is essential to consider species-specific responses (e.g., broilers’ limited thermoregulation vs. swine’s fat insulation), production system variables (intensive vs. semi-intensive), and physiological stages (e.g., early growth, lactation, reproduction), which exhibit differing sensitivities to HS.

Meat quality, including its sensory characteristics and nutritional profile, is closely linked to the health and metabolic status of the animal. HS negatively impacts meat quality through several pathways: it accelerates lipid oxidation, denatures muscle proteins, and disrupts the balance of muscle fibre types, leading to decreased tenderness, altered texture, and reduced water-holding capacity [15]. Acute HS immediately before slaughter increases muscle glycogen breakdown, resulting in pale, soft, and exudative (PSE) meat with poor water-holding capacity. Conversely, prolonged HS reduces muscle glycogen reserves, leading to dark, firm, and dry (DFD) meat with high final pH and increased water retention [7]. Furthermore, HS compromises the nutritional value of meat by degrading essential nutrients such as unsaturated fatty acids, vitamins, and high-quality proteins [16]. Oxidative stress triggered by heat exposure increases lipid peroxidation, which further reduces the shelf life and overall quality of the meat [13]. The economic impact of HS on meat production is substantial, as diminished meat quality lowers market value and increases production costs [17]. Producers are forced to implement costly management strategies, including enhanced cooling systems and dietary modifications, to maintain product quality and consistency.

Effective mitigation strategies are essential to counteract the negative impact of HS on animal health and production efficiency. Nutritional interventions have shown promise in reducing biochemical disturbances caused by HS. Antioxidants like vitamin E, selenium, and flavonoids mitigate oxidative stress by enhancing the activity of enzymes such as superoxide dismutase, catalase, and glutathione peroxidase, which reduce oxidative damage and improve meat quality [18,19]. Osmolytes like betaine maintain cellular hydration and osmotic balance, reducing heat-induced dehydration, improving feed efficiency, and enhancing protein synthesis, leading to better growth performance and meat quality [20]. Probiotics (e.g., *Lactobacillus* and *Bifidobacterium*) and prebiotics (e.g., fructo-oligosaccharides) improve gut health, enhance nutrient absorption, reduce inflammation, and support the gut microbiome, which boosts feed conversion efficiency and carcass traits [19,21]. Phytochemicals such as flavonoids and polyphenols reduce inflammation, preserve muscle structure, and enhance immune function under HS [18]. Minerals like zinc and chromium improve thermoregulation and metabolic balance [22]. Dietary adjustments that increase energy density through fat supplementation help offset reduced feed intake and improve growth performance and feed efficiency [23].

This review examines the complex effects of HS on carcass traits, meat quality, and nutritional value in the key monogastric animals (poultry and swine). It also synthesises current research findings, focusing on how nutritional strategies can mitigate the adverse effects of HS, enhance animal welfare, and promote economic sustainability in monogastric production systems. HS is now recognised as a major environmental challenge, causing significant economic and welfare losses in the poultry and swine sectors [24]. However, despite growing interest in this field, inconsistencies persist in the literature, particularly regarding the effectiveness and reproducibility of nutritional mitigation strategies. Studies often report variable outcomes for antioxidant, electrolyte, and phytochemical supplementation, likely due to differences in species, genetic lines, environmental conditions, and production stages [25]. Moreover, many existing reviews fail to fully integrate species-specific physiological responses with tailored nutritional approaches [26]. To ensure a comprehensive review, an extensive literature search was conducted using databases such as PubMed, Scopus, and Web of Science. Keywords included “heat stress”, “monogastric physiology”, “oxidative stress”, “nutritional interventions”, “meat quality”, “nutritional value”, “antioxidants”, “poultry”, and “swine”. Priority was given to studies published in the last decade, with a focus on insights into molecular mechanisms and nutritional mitigation interventions. The literature was critically analysed to identify gaps in current knowledge and propose integrated solutions for managing HS in monogastric animals.

## 2. Physiological Mechanisms Affecting Carcass Traits, Meat Quality, and Nutritional Value

HS disrupts physiological homeostasis in monogastric animals through multiple mechanisms, most notably by increasing oxidative stress and altering endocrine function. The overproduction of reactive oxygen species (ROS) leads to oxidative damage of lipids, proteins, and cellular structures, impairing muscle integrity and nutrient metabolism [8]. In poultry, this oxidative imbalance is compounded by heat-induced damage to intestinal epithelial cells, resulting in leaky gut and compromised nutrient absorption [27]. Endocrine responses to heat include increased secretion of corticosterone and suppressed thyroid activity, both of which contribute to reduced feed intake, muscle catabolism, and immune suppression [28]. In swine, temperatures exceeding the thermoneutral zone (typically above 27 °C for finishing pigs and sows) lead to significant reductions in growth performance and reproductive efficiency [29]. These losses occur even in the absence of feed intake changes, as HS induces direct metabolic disruptions, including altered nutrient partitioning and reduced lean tissue accretion [29]. These physiological alterations contribute to impaired growth, increased fat deposition, and the deterioration of meat quality in both species.

### 2.1. Muscle Biochemistry Under Heat Stress

Muscle biochemistry is highly sensitive to environmental stressors, particularly heat. Muscle tissue is metabolically active and relies on a fine balance between protein synthesis and degradation to maintain structural integrity, muscle function, and growth. HS disturbs this balance, leading to compromised muscle development, altered muscle fibre composition, and reduced meat quality [30].

Under thermoneutral conditions, muscle protein synthesis and degradation are regulated by a complex network of signalling pathways involving insulin-like growth factor-1 (IGF-1), mammalian target of rapamycin (mTOR), and ubiquitin–proteasome systems [31]. However, exposure to high ambient temperatures reduces the expression of IGF-1 and inhibits mTOR activity, which suppresses protein synthesis and muscle growth.

At the same time, HS activates proteolytic pathways such as the ubiquitin–proteasome system and autophagy, leading to increased protein degradation. This imbalance between reduced synthesis and accelerated degradation results in muscle protein loss, decreased muscle mass, and poor carcass yield [16]. Reduced muscle protein content negatively affects the structural integrity of muscle fibres, leading to tougher meat with lower water-holding capacity and compromised texture.

Protein denaturation is a hallmark of HS-induced muscle damage. High temperatures destabilise the tertiary and quaternary structure of key muscle proteins such as actin and myosin. This loss of structural integrity weakens muscle contraction capacity and reduces *post-mortem* muscle firmness and elasticity [32].

Denatured proteins lose their ability to bind water, reducing the meat’s water-holding capacity and increasing drip loss. This produces drier meat with a tougher texture, negatively impacting consumer satisfaction and market value [15].

HS also alters muscle fibre composition by shifting the balance between oxidative and glycolytic fibres. Oxidative fibres rely on aerobic metabolism and are more resistant to fatigue, whereas glycolytic fibres depend on anaerobic glycolysis for short bursts of activity. Under HS, oxidative fibres decrease while glycolytic fibres increase, resulting in greater reliance on anaerobic metabolism [16]. This shift increases the rate of lactic acid production *post-mortem*, which lowers the pH of muscle tissue and accelerates protein degradation, reducing tenderness and juiciness.

Mitochondria play a central role in muscle energy metabolism through oxidative phosphorylation. HS impairs mitochondrial function by damaging mitochondrial membranes and inhibiting key enzymes involved in the electron transport chain [33]. Reduced oxidative phosphorylation forces muscle cells to rely on anaerobic glycolysis for ATP production. This metabolic shift increases lactic acid accumulation, lowering muscle pH and promoting protein denaturation [31]. Impaired mitochondrial function also increases ROS production, further exacerbating cellular damage and oxidative stress.

### 2.2. Oxidative Stress and Inflammatory Responses

Oxidative stress and inflammation are key drivers of muscle damage and impaired meat quality under HS. Elevated temperatures increase ROS production through mitochondrial dysfunction and the activation of nicotinamide adenine dinucleotide phosphate (NADPH) oxidase. Mitochondrial damage disrupts electron transport chain functioning, increasing electron leakage and the formation of superoxide anions (O_2_^−^), which are converted to hydrogen peroxide (H_2_O_2_) by superoxide dismutase (SOD). In the presence of free iron ions, H_2_O_2_ forms hydroxyl radicals (OH^•^), which cause oxidative damage to lipids, proteins, and DNA [34].

NADPH oxidase further amplifies ROS production by transferring electrons from NADPH to oxygen, increasing oxidative stress and cellular damage. Excess ROS overwhelm antioxidant defences, leading to lipid peroxidation, protein oxidation, and mitochondrial dysfunction [31]. Lipid peroxidation targets unsaturated fatty acids in cell membranes, forming malondialdehyde (MDA) and 4-hydroxynonenal (HNE), which degrade membrane integrity, alter ion balance, and impair nutrient transport. MDA is a widely recognised marker of lipid peroxidation and is commonly used to evaluate oxidative stress in meat tissues under HS conditions [35]. Oxidised myoglobin causes meat discolouration, negatively affecting consumer perception. Damaged mitochondria reduce oxidative phosphorylation efficiency, increasing reliance on anaerobic glycolysis, which promotes lactic acid accumulation, lowers muscle pH, and deteriorates meat texture and tenderness [36].

Oxidative damage to muscle proteins, including actin and myosin, leads to carbonylation and disulfide bond formation, reducing protein functionality and increasing proteolysis. Protein degradation activates the ubiquitin–proteasome system, increasing muscle atrophy and compromising carcass yield. Oxidative stress extends to DNA, causing strand breaks and mutations that impair gene transcription and protein synthesis [34].

ROS-induced damage triggers an inflammatory response by activating nuclear factor-kappa B (NF-κB), which increases pro-inflammatory cytokine production, including tumour necrosis factor-alpha (TNF-α), interleukin-1 beta (IL-1β), and interleukin-6 (IL-6). These cytokines promote further ROS generation, muscle catabolism, and proteolysis [37]. Chronic inflammation increases muscle fibrosis, reduces tenderness, and lowers water-holding capacity, ultimately degrading meat quality and market value. The self-perpetuating cycle of oxidative stress and inflammation underscores the need for targeted nutritional and management strategies to mitigate HS in monogastric animals.

### 2.3. Endocrine Disruptions and Stress Hormone Effects

HS activates the HPA axis, leading to increased secretion of the corticotropin-releasing hormone (CRH) from the hypothalamus. CRH stimulates the pituitary gland to release adrenocorticotropic hormone (ACTH), which, in turn, signals the adrenal cortex to produce cortisol, the primary stress hormone. Elevated cortisol levels play a central role in the metabolic and physiological changes associated with HS, significantly impacting muscle growth, carcass composition, and meat quality [38].

Cortisol inhibits muscle protein synthesis and promotes muscle protein degradation through activation of the ubiquitin–proteasome system. This leads to muscle atrophy and reduced carcass yield. High cortisol levels also increase gluconeogenesis and divert amino acids from muscle protein synthesis toward energy production. Consequently, muscle mass declines while fat deposition increases, altering carcass composition and reducing the proportion of lean meat [7].

HS-induced cortisol elevation disrupts nutrient partitioning by increasing lipogenesis and reducing protein accretion, which contributes to increased fat deposition and marbling. Increased intramuscular fat negatively affects meat texture and taste, reducing overall meat quality. Furthermore, cortisol influences muscle fibre composition by shifting from oxidative (Type I) fibres to glycolytic (Type II) fibres, which reduces muscle endurance and increases susceptibility to lactic acid accumulation *post-mortem*, leading to poor meat texture and tenderness [15].

Cortisol also modulates immune function by suppressing the activity of pro-inflammatory cytokines such as interleukin-6 (IL-6) and tumour necrosis factor-alpha (TNF-α). This immunosuppressive effect increases susceptibility to infections and metabolic disorders, further compromising animal health and productivity [38]. Chronic HS leads to prolonged cortisol elevation, which alters the expression of genes involved in muscle development and metabolism, resulting in long-term reductions in muscle growth and meat quality [39].

The physiological disruptions induced by HS collectively reduce muscle growth, impair carcass composition, and degrade meat quality. These effects highlight the need for targeted nutritional and management strategies to mitigate HS-related losses. Genetic variations in HPA axis responsiveness may also influence an animal’s susceptibility to HS and its associated effects on muscle metabolism and meat quality [38].

## 3. Impact of Heat Stress on Carcass Traits, Meat Quality Attributes, and Nutritional Composition

HS has a profound impact on carcass traits, meat quality, and nutritional composition in monogastric animals, particularly swine and poultry. The adverse effects of HS are primarily driven by disruptions in protein metabolism, oxidative stress, lipid oxidation, and endocrine imbalances, which collectively degrade muscle structure, impair nutrient deposition, and reduce the sensory and nutritional quality of meat. These physiological and biochemical changes have significant implications for the sensory and economic value of meat products, influencing consumer perception and marketability.

In pigs, HS leads to decreased feed intake, resulting in reduced growth performance, lean tissue accretion, and compromised meat quality. The resulting carcasses may exhibit increased fat deposition, lower dressing percentage, and poorer muscle-to-fat ratios. Importantly, the final slaughter weight, often adjusted in response to heat-induced growth delays, plays a significant role in determining carcass traits and market value. Studies have shown that increasing slaughter weight can improve carcass yield, but also leads to greater backfat thickness, which may offset the benefits if market preferences prioritise lean meat [40]. In addition, heavier pigs subjected to HS may be more prone to meat quality defects such as toughness and oxidative instability [36]. Also, in rabbits, divergent selection for body fat content influences meat quality outcomes under HS, as highlighted in studies focusing on oxidative and textural responses to thermal exposure [41]. Thus, aligning slaughter weight with market expectations while considering HS impacts is essential for optimising both meat quality and production efficiency.

### 3.1. Structural and Textural Changes in Muscle Tissue

HS induces significant structural and textural changes in muscle tissue, primarily due to protein denaturation, increased proteolysis, and altered muscle fibre composition. Elevated body temperatures increase metabolic heat production and create an energy deficit, which reduces feed intake and nutrient availability. This metabolic imbalance triggers a shift in muscle metabolism toward catabolism, accelerating muscle protein degradation and impairing protein synthesis [7].

Proteolysis under HS conditions is primarily driven by the activation of the ubiquitin–proteasome system and calpain proteases, which target key structural proteins such as actin and myosin. This degradation reduces muscle firmness and integrity, contributing to muscle shrinkage and increased drip loss [39]. The increased breakdown of myofibrillar proteins leads to the production of peptides and amino acids that further disrupt muscle architecture, resulting in a softer and less cohesive meat texture.

HS also alters muscle fibre composition by increasing the proportion of glycolytic fibres at the expense of oxidative fibres. Glycolytic fibres rely on anaerobic metabolism, which increases lactic acid accumulation *post-mortem*. This accelerates the decline in muscle pH, leading to PSE meat, a common indicator of poor quality in poultry and pork [42]. The reduction in oxidative muscle fibres also lowers myoglobin content, contributing to discolouration and reduced water-holding capacity. Meat from heat-stressed animals tends to lose more water during storage and cooking, reducing both juiciness and tenderness.

The impaired water-holding capacity in heat-stressed meat is linked to protein denaturation and membrane damage caused by oxidative stress. Disruption of the muscle cell membrane reduces the ability of muscle fibres to retain moisture, which increases drip loss and reduces meat yield. Reduced moisture retention also affects the sensory attributes of the meat, making it drier and less tender [16].

### 3.2. Lipid Oxidation and Colour Stability

Lipid oxidation is a major consequence of HS, driven by increased production of ROS and reduced antioxidant defence capacity in muscle tissue. ROS target unsaturated fatty acids in muscle cell membranes, initiating a chain reaction of lipid peroxidation. This process generates cytotoxic by-products such as MDA and HNE, which degrade membrane integrity, impair ion transport, and alter cellular function [35].

Lipid oxidation also affects the colour stability of meat. Myoglobin, the oxygen-binding protein responsible for meat colour, is highly susceptible to oxidative damage. Under HS, myoglobin is rapidly converted from oxymyoglobin (bright red) to metmyoglobin (brown), resulting in discolouration. This reduces the visual appeal of the meat and negatively affects consumer perception [16]. Consumers often associate discolouration with spoilage, even if the meat remains nutritionally sound.

Additionally, increased intramuscular fat deposition under HS conditions makes muscle tissue more vulnerable to lipid oxidation. Nutrient partitioning shifts toward fat storage rather than lean muscle growth, increasing the concentration of polyunsaturated fatty acids (PUFAs), which are more susceptible to oxidative degradation. The breakdown of these fatty acids generates off-flavours and rancidity, reducing the sensory quality of the meat [43].

### 3.3. Sensory Attributes: Tenderness, Juiciness, and Flavour

HS has a profound negative impact on the sensory attributes of meat, including tenderness, juiciness, and flavour. These changes result from biochemical and structural alterations in muscle tissue, including increased proteolysis, altered muscle pH, impaired water-holding capacity, and oxidative damage to lipids and proteins. The combined effects of these changes reduce consumer acceptance and the market value of meat products.

Tenderness is one of the most important sensory attributes influencing meat quality and consumer satisfaction. HS reduces tenderness primarily through increased proteolysis and changes in muscle fibre structure. Under HS conditions, the activation of the ubiquitin–proteasome system and calpain proteases leads to the degradation of key structural proteins such as actin and myosin [39]. These proteins are essential for maintaining the structural integrity and contractile function of muscle fibres. Their degradation weakens the muscle matrix, increasing the susceptibility of muscle fibres to shearing forces and reducing muscle firmness. HS also accelerates *post-mortem* glycolysis due to increased reliance on anaerobic metabolism, leading to the accumulation of lactic acid in muscle tissue. This rapid drop in muscle pH *post-mortem* promotes protein denaturation and the loss of protein solubility, which increases muscle toughness and reduces tenderness [42]. The increased expression of heat shock proteins (HSPs) during HS further interferes with normal protein folding and repair, leading to the accumulation of misfolded proteins, which reduces the elasticity and tenderness of muscle tissue [44]. Additionally, HS alters muscle fibre composition by increasing the proportion of glycolytic fibres, which are less tender and more resistant to degradation than oxidative fibres. The increased proportion of Type II fibres reduces overall tenderness and makes the meat tougher to chew [42]. The loss of muscle protein integrity and increased collagen cross-linking under HS further contribute to reduced tenderness and poor textural quality.

Juiciness is closely linked to the water-holding capacity of muscle fibres, which is impaired under HS. The degradation of structural proteins such as actin and desmin during proteolysis weakens the muscle cell–matrix, reducing the ability of muscle fibres to retain water [39]. Loss of membrane integrity caused by oxidative stress further increases drip loss and reduces water retention during storage and cooking. HS-induced oxidative stress also increases the production of ROS, which damages phospholipids in muscle cell membranes. Lipid peroxidation reduces the fluidity and integrity of muscle membranes, increasing the leakage of intracellular fluids and reducing water-holding capacity [35]. Reduced water-holding capacity leads to increased drip loss during packaging and storage, which reduces both the juiciness and yield of the meat product. Increased lactic acid accumulation *post-mortem* under HS also lowers muscle pH, which causes myosin and actin to denature and aggregate. This reduces the ability of muscle fibres to retain moisture, resulting in drier meat and diminished juiciness [42]. Furthermore, excessive muscle shrinkage caused by increased collagen cross-linking under HS conditions leads to greater fluid loss during cooking, reducing juiciness and making the meat dry and tough.

Flavour is influenced by the composition of fatty acids, amino acids, and volatile compounds in meat. HS reduces flavour quality primarily through lipid oxidation and the breakdown of volatile compounds. Increased oxidative stress during HS promotes the peroxidation of PUFAs in muscle tissue. This generates cytotoxic by-products such as MDA and HNE, which impair membrane integrity and degrade key flavour compounds [35]. Lipid oxidation products such as aldehydes and ketones generate undesirable off-flavours, including rancid, metallic, and grassy notes, which diminish the overall sensory experience [45]. Reduced intramuscular fat content due to impaired nutrient partitioning under HS further limits the availability of flavour precursors, such as oleic acid and linoleic acid, which are essential for the development of a rich flavour profile. HS also alters the amino acid profile of muscle tissue. The breakdown of glutamine and asparagine during increased protein degradation reduces the levels of umami-enhancing compounds, leading to a flatter taste profile. Moreover, the loss of natural antioxidants such as vitamin E and selenium under HS conditions increases susceptibility to further oxidation, compounding flavour degradation [43]. HS also affects the Maillard reaction, which is responsible for the development of complex flavour compounds during cooking. Reduced glycogen content in muscle tissue under HS conditions limits the availability of reducing sugars needed for Maillard browning, leading to a less developed flavour and aroma profile [43].

### 3.4. Nutritional Consequences

HS significantly impairs the nutritional quality of meat by reducing protein content, altering the amino acid and fatty acid profiles, and depleting essential micronutrients. The negative impact on nutrient composition arises from increased metabolic demands, oxidative stress, and protein degradation, which collectively reduce the health benefits and market value of meat products.

HS reduces muscle protein content due to increased proteolysis and impaired protein synthesis. Elevated body temperatures activate the ubiquitin–proteasome system and calpain proteases, which degrade myofibrillar proteins such as actin and myosin [39]. The net result is reduced muscle protein content and impaired muscle growth, which lowers the overall protein value of meat. Increased protein breakdown under HS conditions reduces the concentration of essential amino acids, including lysine, methionine, and threonine, which are critical for muscle repair and human nutrition [7]. Lysine and methionine are particularly important for maintaining muscle integrity and enhancing protein deposition, and their depletion reduces the biological value of meat. Reduced methionine levels also affect sulphur-containing compounds, which play key roles in muscle metabolism and antioxidant defence. Moreover, the amino acid imbalance caused by HS alters the flavour and texture of meat. Reduced glutamine and asparagine levels contribute to a less pronounced umami taste, while increased concentrations of alanine and proline, by-products of muscle breakdown, alter the sweetness and overall taste profile of the meat. The increased activity of heat shock proteins (HSPs) under HS also disrupts protein folding, leading to the accumulation of damaged proteins that are more susceptible to degradation and loss of nutritional quality [42].

HS also compromises the fatty acid composition of meat, particularly by increasing the oxidation of PUFAs. PUFAs, including omega-3 and omega-6 fatty acids, are highly susceptible to peroxidation under oxidative stress. Increased ROS production during HS targets unsaturated fatty acids in muscle cell membranes, initiating lipid peroxidation and forming cytotoxic by-products such as MDA and HNE [35]. The oxidation of omega-3 and omega-6 fatty acids reduces their bioavailability and nutritional value. These fatty acids are essential for maintaining cardiovascular health, regulating inflammatory responses, and supporting cognitive function. Reduced omega-3 content, particularly eicosapentaenoic acid (EPA) and docosahexaenoic acid (DHA), diminishes the anti-inflammatory and heart-protective properties of meat [41]. Increased lipid peroxidation not only reduces the nutritional quality of meat but also generates off-flavours and rancidity, which compromise consumer acceptance. Oxidative damage to intramuscular fat reduces the tenderness and juiciness of meat, and the presence of lipid oxidation by-products such as aldehydes and ketones contributes to undesirable taste and aroma [45]. In addition, HS not only promotes lipid oxidation but also alters the fatty acid composition of muscle tissue, particularly by affecting the enzymatic activity of stearoyl-CoA desaturase (Δ9-desaturase). This enzyme is responsible for converting saturated fatty acids (SFAs), such as stearic acid, into monounsaturated fatty acids (MUFAs), such as oleic acid, which are essential for maintaining membrane fluidity and contributing to meat tenderness and flavour [46]. Under HS conditions, the expression and activity of Δ9-desaturase may be suppressed due to metabolic stress and impaired lipid metabolism, leading to a reduced MUFA/SFA ratio in muscle tissue. This imbalance results in firmer, less flavourful meat due to increased membrane rigidity and lower oxidative stability. In pigs, HS has been shown to significantly decrease Δ9-desaturase activity, as indicated by reduced desaturase indexes, ultimately affecting both carcass traits and meat quality characteristics such as drip loss and intramuscular fat content [43]. Similar findings have shown that HS decreases skeletal muscle metabolic flexibility, including fatty acid oxidation pathways, further supporting the role of lipid metabolism disruption in meat quality deterioration [47]. Furthermore, pork with lower Δ9-desaturase activity tends to show altered fatty acid profiles and poorer sensory attributes such as tenderness and juiciness [48]. The reduction in fat quality and quantity also limits the ability of meat to retain moisture during cooking, further degrading sensory quality.

HS leads to the significant depletion of essential vitamins and minerals, further compromising the nutritional profile of meat. Increased metabolic activity and oxidative stress increase the consumption of endogenous antioxidants such as vitamin E, vitamin C, and glutathione. These antioxidants play a critical role in neutralising ROS and preventing lipid and protein oxidation [45]. In lambs, antioxidant supplementation with selenium and vitamin E has demonstrated improvements in muscle functionality and oxidative stability under HS [45]. Vitamin E, which protects muscle cell membranes from lipid peroxidation, is particularly vulnerable to depletion under HS conditions. Reduced vitamin E levels increase the susceptibility of muscle lipids to oxidative damage, exacerbating rancidity and flavour deterioration [45]. HS also reduces the bioavailability of key trace minerals such as selenium and zinc, which are essential for maintaining antioxidant defence and muscle metabolism. Selenium is a cofactor for glutathione peroxidase, an enzyme that reduces hydrogen peroxide and lipid peroxides. Reduced selenium levels impair the ability of muscle cells to detoxify ROS, increasing oxidative stress and tissue damage. Zinc deficiency further reduces the activity of superoxide dismutase, weakening the antioxidant defence system and increasing the rate of lipid and protein oxidation. B-vitamin levels, particularly riboflavin and niacin, are also reduced under HS. These vitamins play critical roles in energy metabolism and muscle repair. Reduced riboflavin levels limit the efficiency of oxidative phosphorylation, increasing reliance on anaerobic metabolism and lactic acid production. Reduced niacin levels impair the activity of NAD-dependent enzymes, further compromising energy production and muscle function [41].

These changes not only affect the animal, but also have direct implications for consumers. From a consumer standpoint, HS, especially when moderate to severe, has predominantly negative effects on meat quality. It leads to undesirable changes in sensory attributes such as increased toughness, reduced juiciness, and altered flavour due to oxidative damage and disrupted muscle metabolism. Additionally, HS can impair nutritional quality by reducing protein digestibility, essential amino acid availability, and the concentrations of micronutrients such as selenium and vitamin E. While some minor shifts in fatty acid composition have been observed under mild HS, these changes are inconsistent and generally do not translate into clear benefits for consumers. Therefore, HS at any intensity poses challenges for maintaining the quality and appeal of meat products in the consumer market.

Table 1 summarises the key effects of HS on monogastric animals, mainly poultry and swine. The table highlights how HS impacts muscle fibre composition, oxidative stability, and water-holding capacity, ultimately reducing overall meat quality and production efficiency. Understanding these effects is essential for developing effective mitigation strategies to protect animal welfare and maintain product quality under HS conditions.

## 4. Nutritional Strategies to Mitigate Quality and Nutritional Losses

While a variety of strategies, including environmental and physical interventions such as ventilation, cooling systems, and housing modifications, are known to alleviate HS, the focus of this review is specifically on nutritional mitigation strategies. Accordingly, this section explores dietary approaches aimed at counteracting the physiological and carcass-related impacts of HS in monogastric animals.

Nutritional interventions such as antioxidant supplementation and electrolyte balance adjustment can alleviate HS’ effects. However, these interventions should be stage-specific: for example, betaine and vitamin C may be more beneficial during the grower phase in broilers due to high metabolic rates, while gestating sows may benefit from selenium and Vitamin E to maintain oxidative stability and fetal development under heat load. HS disrupts muscle biochemistry, increases oxidative stress, and triggers metabolic and endocrine imbalances, which collectively impair animal performance, compromise meat quality, and reduce the nutritional value of meat products. Targeted dietary strategies aimed at enhancing antioxidant defences, improving metabolic balance, and supporting gut health have proven to be effective in alleviating these negative effects. This section explores key nutritional strategies to mitigate the impact of HS on monogastric animals.

### 4.1. Antioxidant Supplementation

Oxidative stress is a major consequence of HS, driven by the overproduction of ROS, which damages lipids, proteins, and DNA. Excessive ROS overwhelms the animal’s antioxidant defence system, leading to muscle degeneration, poor meat quality, and reduced animal performance [52]. Antioxidant supplementation is a well-established nutritional strategy to counteract oxidative damage and improve muscle integrity and meat quality under HS conditions [53]. Targeted compounds such as vitamin E, vitamin C, and selenium have been shown to enhance antioxidant defences, reduce muscle damage, and support meat quality in heat-stressed poultry [50]. Polyphenols such as resveratrol and curcumin further support thermotolerance by modulating antioxidant enzyme expression and preserving gut integrity [54].

Vitamin E is one of the most potent fat-soluble antioxidants involved in protecting cell membranes from oxidative damage [55]. It functions by scavenging free radicals and interrupting the chain reaction of lipid peroxidation. Vitamin E integrates into the lipid bilayer of muscle cell membranes, enhancing membrane stability and improving the structural integrity of muscle fibres. Supplementing vitamin E has been shown to improve meat quality by enhancing tenderness, juiciness, and colour stability [56]. Additionally, vitamin E protects myoglobin from oxidation, which helps maintain the desirable red colour of fresh meat, a key factor in consumer acceptance.

Selenium is an essential trace element that works synergistically with vitamin E [55]. Selenium is a cofactor for glutathione peroxidase (GPx), an enzyme involved in the detoxification of hydrogen peroxide and lipid peroxides generated during HS. Combined supplementation of vitamin E and selenium has been shown to reduce oxidative damage and enhance muscle integrity and antioxidant capacity [51]. In Japanese quails exposed to HS, a combination of selenium (0.2 mg/kg) and vitamin E (250 mg/kg) resulted in reduced MDA levels, higher SOD activity, and increased serum vitamin E and selenium concentrations [51].

The synergistic effect of vitamin E and selenium has been confirmed in multiple species. In heat-stressed sheep, combined supplementation of selenium (1.2 mg/kg) and vitamin E (100 IU/kg) reduced respiration rate, core body temperature, and oxidative stress biomarkers. Furthermore, supplementation improved feed efficiency and reduced protein degradation under HS conditions.

Polyphenols are secondary plant metabolites with potent antioxidant and anti-inflammatory properties. Studies such as Zeferino et al. [42] have shown that concurrent supplementation of vitamins C and E improves carcass and meat quality traits in broilers exposed to HS. Sources of polyphenols include green tea, grape seed, rosemary, and other herbs [55]. Polyphenols function by scavenging free radicals and inhibiting lipid peroxidation and protein oxidation, which reduces muscle damage and preserves sensory attributes such as tenderness, juiciness, and flavour [57]. Polyphenol supplementation has been shown to reduce oxidative damage to lipids and proteins, lower MDA levels, and improve antioxidant enzyme activity in monogastric animals under HS. For instance, dietary grape seed extract and green tea extract increased catalase and SOD activity while reducing MDA levels in broilers exposed to HS [58]. Polyphenols also inhibit pro-inflammatory cytokines such as tumour necrosis factor-alpha (TNF-α) and interleukin-6 (IL-6), reducing systemic inflammation caused by HS. The anti-inflammatory and antioxidant benefits of polyphenols extend to improved meat quality and shelf life. Supplementation with green tea polyphenols increased water-holding capacity, enhanced juiciness, and reduced drip loss in heat-stressed pigs [57]. These effects translate into improved consumer acceptance and increased economic value of meat products.

Vitamin C, or ascorbic acid, is a water-soluble antioxidant that works alongside vitamin E to protect muscle cells from oxidative stress. Vitamin C directly scavenges ROS and regenerates oxidised vitamin E, maintaining its antioxidant function [59]. Studies have shown that vitamin C supplementation reduces cortisol levels, improves immune function, and enhances feed efficiency in heat-stressed broilers [50].

Other antioxidants, such as carotenoids (e.g., beta-carotene), flavonoids, and lipoic acid, have also shown promise in mitigating the effects of HS. Flavonoids and carotenoids enhance SOD and GPx activity, reduce protein oxidation, and improve muscle colour and texture [57].

The most effective strategy for combating oxidative stress in heat-stressed animals involves combining multiple antioxidants to target different pathways simultaneously. Studies have demonstrated that combining vitamin E, selenium, and polyphenols provides superior protection against oxidative damage than single-agent supplementation [51]. A combined supplementation of vitamin E (250 mg/kg), selenium (0.2 mg/kg), and polyphenols reduced muscle MDA levels by over 30% and increased GPx and SOD activity in broilers under HS [51]. This multifaceted antioxidant approach enhances muscle integrity, improves growth performance, and ensures better sensory attributes in heat-stressed animals.

### 4.2. Osmolytes and Metabolic Modulators

Osmolytes are small organic compounds that stabilise proteins and maintain cellular hydration under HS. They act as molecular chaperones, preserving protein structure, protecting cell membranes, and maintaining ion balance during periods of thermal stress [60]. Osmolytes improve cellular resilience under HS by preventing protein denaturation, maintaining membrane integrity, and enhancing nutrient absorption and metabolic efficiency.

Betaine is one of the most effective osmolytes used in heat-stressed animals [61]. It stabilises protein structures, protects cell membranes, and regulates osmotic balance within muscle cells. Betaine functions as a methyl donor and osmoregulatory molecule, helping cells to maintain hydration and ion balance under thermal stress conditions. The recommended inclusion level of betaine in poultry and swine diets ranges from 0.05% to 0.20% of the total feed, depending on the species, age, and environmental stress levels [62]. Studies have shown that supplementing betaine at 0.12% to 0.20% improves growth performance, increases carcass yield, and reduces abdominal fat deposition in broilers and swine. Betaine supplementation has been shown to improve water-holding capacity, reduce drip loss, and enhance meat tenderness. Additionally, betaine increases the activity of antioxidant enzymes such as SOD and GPx, reducing MDA levels, a marker of oxidative stress [20]. Betaine also enhances feed efficiency and promotes lean muscle growth by improving nutrient absorption and metabolism. It helps reduce the metabolic cost of osmoregulation and supports protein synthesis, which is critical for muscle development and carcass quality under HS conditions. Betaine supplementation at 0.12% has been shown to improve breast meat quality and reduce oxidative stress in broilers under cyclic HS [63].

Taurine is a sulphur-containing amino acid with multifunctional properties, including osmoregulation, antioxidant activity, and membrane stabilisation. Taurine is essential for maintaining cellular osmotic balance, particularly in muscle and cardiac tissues [64]. Its antioxidant properties help protect muscle fibres from oxidative damage caused by increased ROS during HS. The recommended inclusion level of taurine in poultry and swine diets ranges from 0.05% to 0.10% of the total feed. Studies have shown that taurine supplementation at 0.05% to 0.075% significantly improves muscle integrity, reduces drip loss, and enhances meat tenderness in heat-stressed broilers [65]. Taurine supplementation also improved muscle pH, reduced oxidative stress markers (e.g., MDA), and increased the activity of antioxidant enzymes (e.g., SOD and catalase) in heat-stressed animals. Taurine reduces the accumulation of lactic acid in muscle tissue, helping to maintain muscle pH *post-mortem* and preventing excessive protein denaturation. This stabilisation effect improves water-holding capacity and reduces drip loss, key factors in maintaining juiciness and tenderness in heat-stressed animals [66]. Taurine’s ability to regulate calcium homeostasis and improve mitochondrial function also contributes to enhanced muscle recovery and better overall meat quality.

### 4.3. Optimised Diet Formulations

HS reduces feed intake, leading to an energy deficit that accelerates muscle protein degradation and impairs overall growth and meat quality. Reduced feed intake decreases nutrient availability, compromises muscle protein synthesis, and increases metabolic heat production due to the inefficiency of nutrient metabolism under stress [47]. Therefore, optimising diet formulations to increase nutrient density and improve metabolic balance is essential to counteract these effects and maintain production efficiency under HS conditions.

To compensate for reduced feed intake, diets must be reformulated to provide a higher energy density without increasing metabolic heat production. A higher dietary fat-to-protein ratio is often recommended because fat generates less metabolic heat during digestion and metabolism compared to carbohydrates and protein. Increasing dietary fat content while maintaining an optimal balance of essential amino acids improves energy utilisation and supports muscle repair. Formulations with a higher fat-to-protein ratio have been shown to improve growth performance and feed efficiency under HS conditions [67]. A study on broilers demonstrated that increasing dietary energy from 2900 to 3300 kcal/kg with a constant crude protein level of 21% significantly improved feed conversion efficiency, weight gain, and carcass yield under HS [67]. Similarly, a study on dairy cows reported that diets rich in palmitic acid (C16:0) improved fat-corrected milk production and feed efficiency under mild HS, suggesting that high-fat diets can mitigate some of the negative effects of HS. Amino acid balance is also critical. Increased dietary protein levels alone may not be effective unless amino acid profiles are optimised to meet the increased metabolic demands caused by HS. Ensuring an ideal balance of lysine, methionine, and threonine improves protein synthesis and muscle growth under HS. For example, increasing dietary lysine by 0.2% under HS improved feed intake and growth performance in broilers [68] and pigs [69].

Dietary fibres play a crucial role in improving gut health and nutrient absorption under HS. The gut microbiota is highly sensitive to HS, which can disrupt microbial balance and compromise digestive efficiency. Prebiotic fibres, such as fructo-oligosaccharides (FOS) and mannan-oligosaccharides (MOS), promote the growth of beneficial gut bacteria and enhance immune function. A balanced gut microbiome reduces inflammation and improves feed conversion efficiency under HS [70]. FOS supplementation at 0.2–0.4% of feed content has been shown to increase the abundance of *Lactobacillus* and *Bifidobacterium* in the gut, enhancing nutrient absorption and improving gut health in broilers under HS. Improved gut integrity reduces intestinal permeability and prevents the translocation of harmful bacteria, thereby lowering systemic inflammation and improving overall performance [71].

### 4.4. Emerging Feed Additives

New feed additives, including probiotics, prebiotics, and bioactive compounds, have shown potential in improving animal resilience to HS by supporting gut health, metabolic function, and oxidative balance [72]. These additives enhance nutrient absorption, reduce inflammation, and mitigate oxidative stress, thereby improving growth performance and meat quality under HS conditions.

Probiotics, such as *Lactobacillus* and *Bifidobacterium* strains, have been shown to improve gut health, nutrient absorption, and immune function under HS conditions. Probiotics enhance gut barrier integrity by increasing the production of short-chain fatty acids (SCFAs) and reducing intestinal permeability. They also inhibit the growth of pathogenic bacteria and modulate the immune response, reducing inflammation and oxidative stress [70].

Prebiotics, such as inulin and FOS, provide a substrate for beneficial gut bacteria, improving gut microbiota balance and enhancing feed conversion efficiency. A study on broilers showed that supplementation with FOS at 0.3% improved gut integrity, increased nutrient absorption, and enhanced growth performance under HS [70].

Phytochemicals such as flavonoids and polyphenols modulate key metabolic pathways involved in muscle protein synthesis and degradation. They reduce oxidative stress, inhibit inflammation, and enhance muscle fibre integrity. Polyphenol-rich extracts from grape seed and green tea have been shown to reduce MDA levels and increase the activity of antioxidant enzymes such as SOD and catalase in broilers under HS [58].

### 4.5. Integrative Approaches

While individual nutritional strategies have demonstrated benefits in mitigating the effects of HS, combining multiple approaches often produces the most significant improvements. An integrative strategy that combines antioxidants (e.g., vitamin E, selenium), osmolytes (e.g., betaine, taurine), probiotics, and phytochemicals creates a synergistic effect that addresses multiple physiological challenges simultaneously [73]. By targeting oxidative stress, metabolic imbalances, and gut health concurrently, integrated nutritional strategies enhance muscle integrity, improve carcass traits, and sustain the nutritional value of meat.

An example of the success of an integrative strategy comes from a study on lactating sows under HS. Liu et al. (2017) [74] demonstrated that a combined supplementation of selenium (0.4 ppm), vitamin E (95 IU/kg), chromium (0.4 ppm), and betaine (0.2%) significantly reduced body weight loss and improved lactational performance without affecting feed intake. The combined supplementation also enhanced feed efficiency, reduced oxidative stress, and improved immune function. The synergistic effect of selenium and vitamin E improved antioxidant capacity by increasing glutathione peroxidase activity and reducing MDA levels, a marker of oxidative stress. Meanwhile, betaine improved nutrient absorption and protein synthesis, contributing to better muscle growth and improved meat quality [74].

Another example comes from poultry production. Hajati et al. (2018) [58] investigated the effects of combining grape seed extract (a rich source of polyphenols) with vitamin E and selenium in broiler diets under HS. The combined supplementation significantly increased antioxidant enzyme activity (superoxide dismutase and glutathione peroxidase), reduced MDA levels, and enhanced oxidative stability in breast muscle. The combined strategy also improved sensory attributes, including meat tenderness and juiciness, while reducing drip loss and improving water-holding capacity [58]. The polyphenols from grape seed extract acted synergistically with vitamin E and selenium, enhancing the overall antioxidant capacity and preserving muscle integrity under HS.

Similarly, studies in broilers have shown that combining selenium (0.2 mg/kg) and vitamin E (250 mg/kg) enhances oxidative stability and reduces muscle damage under HS. The inclusion of betaine and taurine in the diet further improves water-holding capacity and muscle pH, reducing drip loss and enhancing meat tenderness [66].

Table 2 summarises these nutritional strategies, including feed additives, antioxidants, and electrolyte supplements that have shown promise in maintaining growth performance and meat quality under thermal stress.

## 5. Practical Implications and Economic Considerations

The adverse impacts of HS on meat quality and nutritional value have far-reaching practical and economic implications for monogastric livestock production, mainly poultry and swine. HS reduces animal welfare, compromises production efficiency, and increases the economic burden on producers due to lower carcass yields, reduced product quality, and increased production costs. In poultry, acute HS during the final grow-out phase predominantly affects breast muscle quality, while in swine, chronic HS during gestation can impair reproductive performance and offspring viability. These differences necessitate tailored interventions by species and stage. Addressing these challenges requires a holistic approach that combines nutritional interventions with improved management practices and infrastructural investments [16]. Effective strategies for HS management not only enhance animal performance but also preserve the economic viability of production systems.

### 5.1. Management and Nutritional Adaptations

Implementing nutritional interventions to mitigate HS requires significant adaptations in feed manufacturing and dietary planning. Feed formulations must be modified to include additional supplements such as antioxidants (e.g., vitamin E, selenium), osmolytes (e.g., betaine, taurine), and bioactive feed additives (e.g., polyphenols, probiotics). These changes aim to improve cellular integrity, reduce oxidative damage, and enhance metabolic efficiency under thermal stress [75].

While these dietary modifications may initially increase production costs, the long-term benefits, such as enhanced animal performance, improved carcass yield, and higher meat quality, can offset these expenses. For instance, combining selenium and vitamin E supplementation has been shown to reduce oxidative stress, improve feed efficiency, and enhance muscle integrity in heat-stressed broilers [7]. Furthermore, dietary betaine at inclusion levels of 0.12–0.20% has been shown to improve water-holding capacity, reduce drip loss, and enhance overall meat tenderness in both poultry and swine [66].

In addition to dietary changes, improvements in environmental management are critical for reducing the thermal load on animals. Investments in housing design, ventilation, and cooling technologies can work synergistically with dietary strategies to reduce the thermal load on animals. For example, installing evaporative cooling pads and automated ventilation systems can reduce the effective temperature by 4–8 °C, improving animal comfort and reducing heat-related mortality rates [15]. The use of natural shading from trees or constructed shade structures has also demonstrated positive effects on animal welfare and productivity.

Species-specific approaches are also essential. Poultry and swine exhibit different physiological responses to HS, necessitating tailored strategies. Poultry are particularly sensitive to sudden heat waves due to their limited ability to dissipate heat. Rapid dietary adjustments, such as increasing the dietary inclusion of vitamin C (200–500 mg/kg) and betaine (0.12–0.20%), have been shown to improve feed efficiency and carcass yield during periods of acute HS [50]. In contrast, swine are more susceptible to chronic thermal stress, which necessitates long-term nutritional adjustments. The inclusion of selenium (0.2–0.4 mg/kg) and polyphenols (100–300 mg/kg) has been shown to improve antioxidant capacity, reduce oxidative stress markers, and enhance muscle integrity in pigs under prolonged HS [58]. Tailoring interventions to the specific needs of each species is essential for maximising the effectiveness of HS mitigation strategies. On-farm trials and continuous monitoring are critical to fine-tuning these strategies and ensuring that they meet the unique metabolic and physiological requirements of each production system.

### 5.2. Economic and Market Implications

Economic losses from HS in monogastric production systems are substantial, with estimates varying based on species, production systems, and regional environmental conditions. For swine production, HS decreases feed efficiency, increases fat deposition, and reduces lean muscle yield, leading to carcass quality deterioration and lower market value [29]. In swine, postnatal HS can reduce growth rates by up to 15% and increase fat-to-muscle ratios by 5–8%, decreasing carcass value by approximately USD 10–20 per animal.

In poultry, HS has been linked to a 5–12% reduction in carcass weight, reduced breast meat yield, and increased incidence of PSE meat [15]. This reduction in product quality leads to lower market prices and increased processing costs. Broiler producers face additional costs for cooling systems, estimated at USD 0.08–0.12 per bird, to offset HS effects. Overall, the poultry sector faces global economic losses from HS exceeding USD 2 billion annually, with significant impacts in regions with high ambient temperatures [76].

### 5.3. Policy and Financial Incentives

Given the significant challenges posed by HS, policymakers and industry stakeholders have an important role in supporting mitigation efforts. Financial incentives, such as subsidies, tax breaks, or technical support programmes, can encourage producers to invest in improved nutritional strategies and modern housing technologies. Such policy measures not only enhance the resilience of meat production systems but also contribute to broader goals of sustainable agriculture and food security. By facilitating the adoption of integrated approaches to HS mitigation, policymakers can help ensure the long-term viability of the livestock industry in the face of climate change [77].

To ensure the economic viability of HS mitigation strategies, comprehensive on-farm cost–benefit analyses are essential. These evaluations should consider the initial costs of implementing nutritional interventions and environmental improvements, as well as the long-term benefits in terms of improved animal performance, higher carcass yields, and enhanced meat quality. Long-term studies that assess the cumulative effects of these interventions across multiple production cycles will provide valuable insights into their overall economic impact. Such evaluations are critical for guiding decision-making and encouraging the widespread adoption of integrated HS mitigation strategies.

## 6. Conclusions and Future Perspectives

HS significantly impairs carcass traits, meat quality, and nutritional composition in monogastric animals, particularly poultry and swine, posing major challenges to animal welfare, production efficiency, and economic viability. From the consumer perspective, moderate to severe HS predominantly degrades meat quality, affecting sensory attributes such as tenderness, juiciness, and flavour, as well as nutritional value. These effects are driven by HS-induced disruptions in protein metabolism, mitochondrial function, muscle fibre structure, and endocrine balance, which collectively lead to oxidative stress, lipid peroxidation, inflammation, and tissue degradation.

Targeted nutritional interventions, including supplementation with antioxidants (e.g., vitamin E, selenium, polyphenols), osmolytes (e.g., betaine, taurine), probiotics, prebiotics, and balanced dietary formulations, have demonstrated promising outcomes in mitigating these adverse effects. In particular, emerging additives such as phytochemicals offer additional protection by enhancing gut integrity, immune function, and thermotolerance. When combined with environmental control strategies (e.g., ventilation, cooling systems), these dietary approaches contribute to a comprehensive and sustainable HS mitigation framework.

To maximise efficacy, nutritional strategies must be tailored to species, production stage, and production system. Future research should prioritise precision nutrition, validated biomarkers, and long-term multi-factorial studies. Additionally, the integration of genetic selection for heat tolerance with nutritional and environmental management holds great promise for improving resilience.

Policymakers and industry stakeholders should promote the implementation of integrated HS mitigation strategies through targeted incentives, research investment, and knowledge transfer. Continued collaboration across scientific, regulatory, and industry sectors is essential to ensure the long-term sustainability, productivity, and welfare of monogastric livestock systems in an increasingly warm climate.

## Figures and Tables

**Table 1:** Effects of heat stress on meat quality and composition in monogastric animals.

Impact Area	Mechanism	Effects on Meat Quality and Composition	Physiological Consequences	Economic and Consumer Impact	References
Structural and Textural Changes	- Protein denaturation due to heat-induced instability - Increased proteolysis via calpain and ubiquitin–proteasome pathways - Shift from oxidative to glycolytic fibres - Mitochondrial dysfunction reduces ATP availability	- Reduced tenderness and muscle firmness - Increased drip loss - Rapid *post-mortem* pH decline, causing PSE meat - Lower muscle fibre density	- Reduced muscle mass - Increased muscle fragility	- Lower market value - Increased processing loss	[15,16]
Lipid Oxidation and Colour Stability	- Increased ROS production targets unsaturated fatty acids - Lipid peroxidation generates MDA and HNE - Myoglobin oxidation leads to colour changes	- Loss of membrane integrity - Reduced oxidative stability - Discoloration due to myoglobin oxidation	- Reduced cell viability - Increased lipid rancidity	- Reduced consumer appeal - Shorter shelf life	[35,49]
Sensory Attributes (Tenderness, Juiciness, Flavour)	- Increased proteolysis of actin and myosin - Lactic acid accumulation lowers muscle pH - Lipid oxidation produces aldehydes and ketones	- Reduced tenderness and juiciness - Increased off-flavours - Lower water-holding capacity	- Poor muscle texture and flavour profile	- Lower consumer satisfaction - Negative product perception	[16,42]
Protein Content and Amino Acid Profile	- Increased muscle proteolysis - Reduced protein synthesis due to impaired mitochondrial function - Increased muscle catabolism	- Reduced muscle protein content - Loss of essential amino acids	- Reduced muscle growth and repair	- Lower market value - Lower biological value	[7]
Fatty Acid Composition and Lipid Quality	- Oxidation of PUFAs - Increased lipid peroxidation due to ROS production	- Lower omega-3 and omega-6 content - Increased rancidity and off-flavours	- Reduced lipid reserves - Increased oxidative damage	- Lower nutritional value - Shorter shelf life	[41,42]
Micronutrient Degradation	- Depletion of vitamins and antioxidants - Increased ROS-induced damage to membranes - Loss of essential trace minerals	- Reduced shelf life and oxidative stability - Lower antioxidant capacity	- Increased muscle susceptibility to oxidative stress	- Reduced nutritional value - Lower consumer preference	[50,51]
Endocrine Disruptions	- Activation of the HPA axis increases cortisol levels - Cortisol promotes muscle degradation and increases fat deposition	- Reduced muscle mass - Increased fat deposition	- Reduced muscle growth - Increased fat accumulation	- Lower carcass yield - Reduced market value	[38,42]
Consumer and Economic Implications	- Reduced sensory and nutritional quality - Increased production costs due to heat-stress management	- Lower consumer acceptance due to poor texture and flavour - Reduced shelf life	- Increased production costs - Lower profitability	- Lower market value - Reduced profitability	[43]

**Table 2:** Major nutritional strategies to mitigate the effects of heat stress on monogastric animals.

Nutritional Strategy	Mechanism of Action	Animal Species (Start–End Duration)	Inclusion Levels	Effects on Heat Stress	References
Vitamin E	Fat-soluble antioxidant; integrates into cell membranes and scavenges free radicals	Broilers (1–35 days); Pigs (25–70 kg)	100–250 IU/kg	Improves tenderness, juiciness, and colour stability; reduces lipid oxidation	[51,56]
Selenium	Cofactor for glutathione peroxidase; detoxifies hydrogen peroxide and lipid peroxides	Broilers (7–35 days); Pigs (20–70 kg); Quails (5–20 days)	0.2–0.4 mg/kg	Enhances antioxidant capacity, reduces oxidative stress, and improves muscle integrity	[49,51]
Polyphenols	Scavenges free radicals, inhibits lipid peroxidation, and reduces pro-inflammatory cytokines	Broilers (14–49 days)	100–300 mg/kg from grape seed extract or green tea	Improves tenderness, juiciness, colour stability, and shelf life	[58]
Betaine	Osmolyte; maintains cellular hydration and ion balance, stabilizes proteins	Broilers (14–42 days); Pigs (20–70 kg)	0.12–0.20% of diet	Improves water-holding capacity, reduces drip loss, and enhances feed efficiency	[62,63]
Taurine	Osmolyte and antioxidant; stabilizes cell membranes and reduces lactic acid accumulation	Broilers (14–42 days); Pigs (20–65 kg)	0.05–0.10% of diet	Improves muscle integrity, reduces drip loss, and enhances tenderness	[65,66]
Vitamin C	Water-soluble antioxidant; scavenges ROS and regenerates oxidized vitamin E	Broilers (14–49 days); Pigs (20–70 kg)	200–500 mg/kg	Reduces cortisol levels, enhances feed efficiency, and improves immune function	[50]
Probiotics	Beneficial microorganisms (e.g., Lactobacillus, Bifidobacterium) that enhance gut health	Broilers (7–35 days); Pigs (20–65 kg)	10^7^–10^9^ CFU/kg	Improves nutrient absorption, reduces inflammation, and increases feed efficiency	[70]
Prebiotics	Non-digestible fibres (e.g., FOS, MOS) that promote the growth of beneficial gut bacteria	Broilers (7–35 days); Pigs (20–65 kg)	0.2–0.4% of diet	Improves gut microbiota balance, reduces inflammation, and increases feed efficiency	[70]
Phytochemicals	Bioactive compounds (e.g., flavonoids, terpenoids) that modulate oxidative and inflammatory responses	Broilers (14–49 days)	100–300 mg/kg	Reduces oxidative stress, improves muscle integrity, and enhances sensory attributes	[57]
Optimized protein-to-energy ratio	Adjusts dietary protein and fat levels to improve energy balance under reduced feed intake	Broilers (7–35 days); Pigs (20–70 kg)	2900–3300 kcal/kg with 20–22% protein	Improves growth performance and carcass yield	[67]
Combination of Vitamin E, Selenium, and Betaine	Synergistic antioxidant and osmoprotective effects	Broilers (14–42 days); Sows (40–120 kg)	Vitamin E (250 IU/kg), Selenium (0.2 mg/kg), Betaine (0.2%)	Enhances antioxidant capacity, improves muscle performance, and reduces oxidative damage	[74]
Combination of Vitamin E, Selenium, and Polyphenols	Synergistic antioxidant and anti-inflammatory effects	Broilers (14–49 days)	Vitamin E (250 mg/kg), Selenium (0.2 mg/kg), Polyphenols (100–300 mg/kg)	Improves oxidative stability, reduces drip loss, and enhances sensory traits	[58]

## Data Availability

No new data were created or analyzed in this study. Data sharing is not applicable to this article.
